# Early functional rehabilitation versus traditional immobilization for surgical Achilles tendon repair after acute rupture: a systematic review of overlapping meta-analyses

**DOI:** 10.1038/srep39871

**Published:** 2017-01-05

**Authors:** Jia-Guo Zhao, Xiao-Hui Meng, Lin Liu, Xian-Tie Zeng, Shi-Lian Kan

**Affiliations:** 1 Department of Orthopaedic Surgery, Tianjin Hospital, Tianjin, China; 2 Department of Orthopaedic Surgery, Yixing Traditional Chinese Medicine Hospital, Jiangsu Province, China; 3 Graduate School, Tianjin University of Traditional Chinese Medicine, Tianjin, China

## Abstract

Several meta-analyses comparing early functional rehabilitation and traditional immobilization following surgical Achilles tendon repair after acute rupture have been published. However, they have led to conflicting conclusions. The aims of this systematic review were to select high-quality meta-analyses from multiple discordant meta-analyses and to provide a postoperative rehabilitation strategy following surgical repair using currently available evidence. We performed a comprehensive search using the PubMed and Embase databases and the Cochrane Library. Assessment of Multiple Systematic Reviews (AMSTAR) instrument was used to assess the methodological quality. Three investigators independently applied the Jadad decision algorithm. Their results were then compared to ensure selection of a meta-analysis that provided the highest quality of evidence. Six meta-analyses met the eligibility criteria. AMSTAR scores ranged from 6 to 10. According to the Jadad decision algorithm, a high-quality meta-analysis with a greater number of RCTs was selected. This meta-analysis showed that early functional rehabilitation was superior to cast immobilization in terms of patient satisfaction and the time to return to pre-morbid sporting levels. There were no differences regarding major complications or the time before return to prior employment and sporting activity. Thus, we recommend early functional rehabilitation as the postoperative strategy for acute Achilles tendon ruptures.

Ruptures of the Achilles tendon are common, with an overall incidence of 18 per 100,000 per year^1^. Although conservative treatment has its advantages, surgical intervention appears to be the available method for athletes and young people^2^. Traditionally, surgical management of acute Achilles tendon rupture was combined with ankle immobilization for 6 weeks. This remained the standard approach until the late 1980s^2^,^3^,^4^.

Postoperative functional rehabilitation after Achilles tendon repair has been increasingly discussed during recent years^5^. The traditional rehabilitation protocol has involved rigid cast immobilization, usually in a below-knee non-weight bearing rigid cast for six weeks, followed by mobilization of the ankle joint and strengthening exercises^2^,^3^. However, initial clinical trials^6^,^7^ using early postoperative ankle mobilization and functional rehabilitation showed a low rate of re-rupture. Subsequently, multiple authors reported numerous randomized controlled trials (RCTs)^7^,^8^,^9^,^10^,^11^,^12^,^13^ to compare early functional rehabilitation with cast immobilization. Based on the proliferation of RCTs, several systematic reviews and meta-analyses^14^,^15^,^16^ were published. However, they have led to conflicting conclusions. The best postoperative rehabilitation strategy for acute Achilles tendon rupture remains a topic of debate.

The aims of this systematic review were as follows: (1) to conduct a systematic review of meta-analyses comparing early functional rehabilitation and traditional immobilization following surgical Achilles tendon repair after acute rupture; (2) to select high-quality meta-analyses among multiple discordant meta-analyses; and (3) to provide a postoperative rehabilitation strategy following surgical repair using currently available evidence.

## Materials and Methods

We performed this systematic review in accordance with the PRISMA guidelines^17^.

### Literature Search

We conducted a comprehensive search using the PubMed and Embase databases and the Cochrane Library. The following keywords were used: ((((Achilles tendon) OR Achilles) OR tendoachilles)) AND ((((rupture) OR injury) OR lesion) OR tear). We limited the article types to meta-analyses or systematic reviews. The selected articles were from the English literature. The search was performed on December 27, 2015. The references of the included studies were also manually searched such that no meta-analyses were missed. Furthermore, we manually searched the following journal contents from the past 3 years for any additional studies: *The Journal of Bone and Joint Surgery, The British Journal of Sports Medicine, The Bone and Joint Journal,* and *The American Journal of Sports Medicine.*

### Eligibility Criteria

We identified meta-analyses/systematic reviews comparing early functional rehabilitation with traditional immobilization following surgical Achilles tendon repair after acute rupture. The exclusion criteria were as follows: (1) narrative review without a reported and organized search algorithm; (2) meta-analysis that included non-RCTs; (3) systematic review that did not perform a meta-analysis; and (4) meta-analysis without clinical outcome data.

### Selection of Studies

Full-text articles for studies meeting eligibility criteria were selected. Two investigators independently extracted information from each included study. The following information was extracted for the included meta-analyses: journal of publication, levels of evidence, primary author, date of literature search and publication, eligibility criteria, search database, design of primary studies, number of primary RCTs, software use, performance of heterogeneity analysis, sensitivity or subgroup analysis and conflicts of interest. Outcome data were also extracted, such as ankle function, patient satisfaction and adverse events.

### Assessment of Methodological Quality

Two authors independently assessed the methodological quality of the included studies. We assessed the risk of bias using the Assessment of Multiple Systematic Reviews (AMSTAR) instrument. This instrument provides 11 categories for evaluating meta-analyses/systematic reviews according to the quality of their reporting and methodology^18^. We also graded meta-analysis quality using The Oxford Evidence-based Medicine Levels of Evidence^19^.

### Application of the Jadad Decision Algorithm

We interpreted and selected the discordant meta-analyses using the Jadad decision algorithm^20^, which is a useful tool to differentiate overlapping systematic reviews/meta-analyses. The Jadad Decision Algorithm^20^ was designed based on following questions: (1) Do the meta-analyses ask the same question? (2) Do the meta-analyses include the same studies? (3) Do the meta-analyses containing the same trials have the same methodologic quality? (4) Do the discordant meta-analyses including different trials use the same selection criteria^20^? Three investigators independently applied this algorithm, and their results were then compared to ensure selection of the meta-analysis that provided the highest quality of evidence to develop recommendations for a postoperative rehabilitation strategy after surgical repair.

## Results

### Search Results

**Figure 1:**
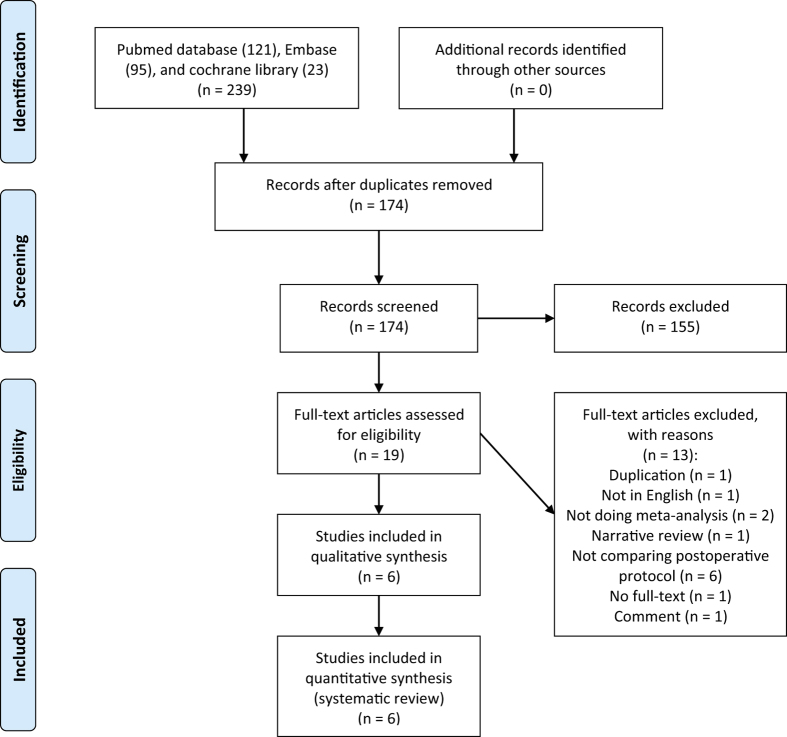
Flow diagram summarizing the selection process of meta-analyses.

We initially identified 274 abstracts. Six studies^14^,^15^,^16^,^21^,^22^,^23^ met the eligibility criteria and were included. A flow diagram is shown in Fig. 1. These studies were published between 2004 and 2016. Three^15^,^21^,^23^ of them were published within the past two years (Table 1). All of the studies performed a meta-analysis. Thirteen RCTs were included in the various meta-analyses. The number of trials cited in the meta-analyses ranged from 5 to 10, with a median of seven primary RCTs cited (Table 2). The number of included patients ranged from 273 to 570, with an average of 377 patients per study.

### Search Methodology

All studies comprehensively searched databases. Each meta-analysis used at least four different databases to research RCTs. All studies included meta-analyses searched from PubMed or Medline, Embase and the Cochrane Library. There was heterogeneity among the other databases that were used. Details of the search methodology are summarized in Table 3.

### Study Quality and Validity

Only RCTs were included for all meta-analyses. The AMSTAR scores were assessed for the included meta-analyses and ranged from 6 to 10 points, with a median of 7.3 points (with the maximum possible score of 11 points). One Cochrane Review^22^ was assessed as the best-quality study. The AMSTAR score for each meta-analysis is shown in Table 4. According to the Oxford Levels of Evidence, three meta-analyses^15^,^16^,^22^ were assessed as Level I evidence, and four studies^14^,^21^,^23^ were assessed as Level II evidence (Table 5).

### Heterogeneity Assessment

Statistical heterogeneity was assessed in each included meta-analysis using the I^2^ statistic value. Revman software was used for all meta-analyses (Table 5). Only one meta-analysis^23^ performed subgroup analyses. Five other meta-analyses^14^,^15^,^16^,^21^,^22^ did not perform sensitivity or subgroup analyses (Table 5).

### Results of the Jadad Decision Algorithm

**Figure 2:**
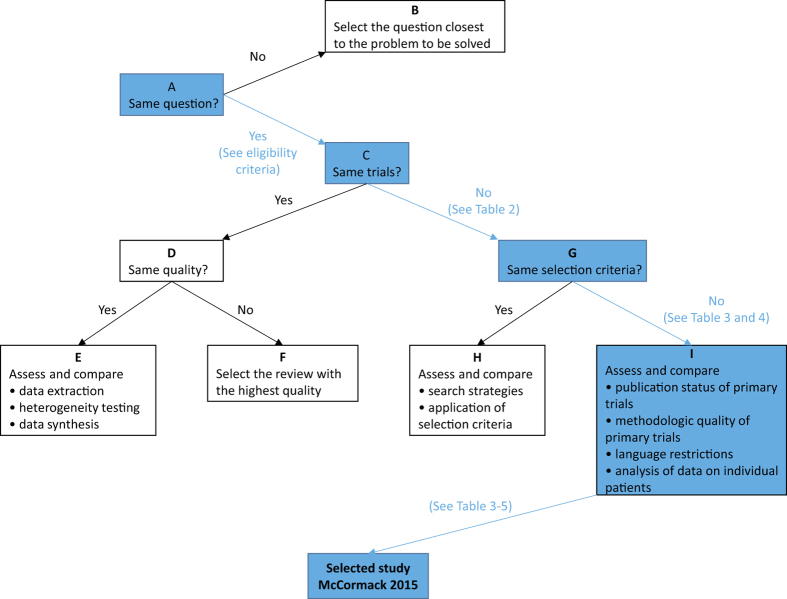
Flow diagram of the Jadad decision algorithm.

Three authors independently selected the same flow path according to the Jadad decision algorithm. Because (1) all included meta-analyses analysed the same question (comparing early functional rehabilitation with traditional immobilization following surgical Achilles tendon repair after acute rupture), (2) the included meta-analyses did not include the same RCTs, and (3) they did not have the same selection criteria, we selected the highest-quality meta-analysis based on the following factors: publication status of the primary studies, methodology of the primary studies, language restrictions and the analysis of data on individual patients. None of the eligible meta-analyses stated a restriction on the publication status of primary studies. Regarding the methodology of primary studies, all meta-analyses exclusively included RCTs with superior methodologies. McCormack and Bovard^15^ restricted the study quality using a minimum of 19 items of the Downs and Black-validated 27-item checklist^24^. Concerning the publication characteristics, the included meta-analyses were published over an extended period. Thus, more recent meta-analyses were preferred to less recent meta-analyses. Additionally, all meta-analyses appropriately analysed the data. Finally, three authors selected the study by McCormack and Bovard^15^ as the meta-analysis offering the best current evidence (Fig. 2).

McCormack and Bovard^15^ reported that early functional rehabilitation was superior to cast immobilization regarding patient satisfaction and the time interval of return to prior sporting level. Furthermore, they found no differences concerning major complications and the rate of return to prior employment and sporting activity in their study. Hence, they concluded that early functional rehabilitation following surgical repair of acute Achilles tendon rupture was safe and leaded to higher patient satisfaction and earlier return to function.

## Discussion

Multiple RCTs^9^,^10^,^11^,^12^,^13^ comparing early functional rehabilitation with cast immobilization have been reported. However, they have presented conflicting findings regarding which strategy is better. Furthermore, many meta-analyses of RCTs, representing the highest grade of evidence, have been reported to compare two postoperative rehabilitation strategies. Although three meta-analyses^15^,^21^,^23^ comprehensively searched databases during the most recent two years, they included different RCTs and reached conflicting results. Such discordance has confused decision makers (such as policymakers, clinicians and patients) who make choices based on these meta-analyses.

The possible sources of discordant results among meta-analyses were analysed and reported by Jadad *et al*.^20^ Meanwhile, they designed a decision tool to choose high-quality evidence among discordant systematic reviews. This decision tool has been widely used to find the best available evidence among overlapping systematic reviews/meta-analyses^25^,^26^. Khan *et al*.^22^ performed a Cochrane systematic review comparing early functional rehabilitation with immobilization after surgical Achilles tendon repair. The Cochrane systematic review is internationally recognized as “the highest standard” in evidence-based health care resources and should be updated every two years to provide the most current evidence to decision makers. The Cochrane review by Khan *et al*.^22^ obtained the highest scores based on the AMSTAR instrument in the current study. However, their review was performed in 2004 and was not updated for 13 years regarding the postoperative strategy, although multiple newly available RCTs have emerged. Hence, we did not select Khan *et al*.^22^ as the best review. McCormack and Bovard^15^ included the maximum number of RCTs and appropriately analysed the RCT data. Hence, the McCormack and Bovard^15^ meta-analysis was selected to represent the best available current evidence.

McCormack and Bovard^15^ found that the early rehabilitation group had three times the odds of rating patient satisfaction as ‘good’ or ‘excellent’ compared to the traditional immobilization group (p = 0.01; OR, 3.13; 95% CI 1.30 to 7.53). They also reported that there were no differences concerning major complications (including tendon re-rupture, deep infection, tendon adhesions, persistent functional/neurological deficit, thrombophlebitis/compartment syndrome and wound slough) or the rate of return to prior employment and sporting activity. However, five of the six included RCTs showed that the early rehabilitation group had a shorter time interval before return to prior sporting level than the traditional immobilization group. They concluded that early functional rehabilitation resulted in higher patient satisfaction and earlier return to function, with no increase in complications^15^. Thus, early functional rehabilitation may contribute to evidence-based practice for postoperative management after Achilles tendon repair.

The phases of tendon healing have been divided into inflammation, proliferation and remodelling^5^. Throughout these phases, the tensile strength of the tendon gradually increases, although it remains inferior to the uninjured tissue. The new scar tissue is biomechanically inferior, which causes increased stiffness and subsequent visco-elastic properties^27^. Animal studies have demonstrated that early rehabilitation can decrease excessive adhesion formation, improve biomechanical properties of the scar tissue and subsequently enhance the gliding function of the tendon^28^,^29^. Active or passive ankle joint motion exercises can eliminate local oedema and prevent joint stiffness and the atrophy of calf muscles^8^. Re-rupture following surgical repair is a potential complication for early function. Many surgeons worry that early function may increase the re-rupture rate. However, the results of the current study demonstrate that early functional rehabilitation after Achilles tendon repair does not increase the re-rupture rate and also improves patient satisfaction and facilitates earlier return to activity.

Our study has several strengths. First, this study can be considered as the first systematic review of overlapping meta-analyses to evaluate the rehabilitation strategy following surgical management of acute Achilles tendon rupture. Second, our study provides high-quality evidence for a postoperative rehabilitation strategy following surgical repair based on comprehensive summarization of previously published meta-analyses on this topic. Our study is a systematic review of overlapping meta-analyses, which is used to appraise the methodological quality and quality of reporting of meta-analyses. It is different from traditional systematic reviews that analyse primary studies. In this study, we selected the best of the currently published meta-analyses and provided high-level evidence to decision makers. Hence, the results of our study have greater strength than other systematic reviews or RCTs.

There are several limitations to this systematic review. First, this systematic review was not registered on prospective registration systems for systematic reviews. Prospective registration could improve the quality of a systematic review and increase confidence in the findings. Second, the AMSTAR tool showed that the methodological quality of the included meta-analyses was low. Although we only assessed meta-analyses, including RCTs, to ensure high methodological quality, three of the studies were assessed as Level II evidence. Most included meta-analyses did not provide a priori design. Furthermore, none of them assessed the likelihood of publication bias. Third, only English language studies were researched in this systematic review. Our search strategy may have excluded non-English studies meeting eligibility criteria. Fourth, there was a clear lack of blinding in all trials, which may have influenced patient-reported outcome measures, such as patient satisfaction. However, blinding was not possible for participants and clinicians because of the nature of surgical interventions. Fifth, the surgical techniques varied between trials, which also might have presented significant residual confounding. However, we also noted that several RCTs reported no significant differences in postoperative outcomes between traditional open and minimally invasive surgery^30^,^31^,^32^.

In brief, this systematic review of meta-analyses concludes that early functional rehabilitation can improve patient satisfaction and facilitate earlier return to activity without increased complication rates. Thus, we recommend early functional rehabilitation as the postoperative strategy for acute Achilles tendon ruptures.

## Additional Information

**How to cite this article**: Zhao, J.-G. *et al*. Early functional rehabilitation versus traditional immobilization for surgical Achilles tendon repair after acute rupture: a systematic review of overlapping meta-analyses. *Sci. Rep.* **7**, 39871; doi: 10.1038/srep39871 (2017).

**Publisher's note:** Springer Nature remains neutral with regard to jurisdictional claims in published maps and institutional affiliations.

## Figures and Tables

**Table 1:** General description of the characteristics of each meta-analysis.

Author	Journal Name	Date of Last Literature Search	Date of Publication	No. of Included Trials	No. of Included RCTs
Khan 2004	*Cochrane Database of Systematic Reviews*	September 2003	July 2004	5	5
Khan 2005	*Journal of Bone and Joint Surgery Am*	NA	October 2005	5	5
Suchak 2006	*Clinical Orthopaedics and Related Research*	July 2004	April 2006	6	6
Mark-Christensen 2016	*Knee Surgery, Sports Traumatology, Arthroscopy*	May 2013	June 2016	7	7
Huang 2015	*The American Journal of Sports Medicine*	August 2013	April 2015	9	9
McCormack 2015	*The British Journal of Sports Medicine*	June 2015	October 2015	10	10

**Table 2:** Primary studies included in meta-analyses.

Author/Year	Saleh 1992	Cetti 1994	Mortensen 1999	Kerkhoffs 2002	Costa 2003	Kangas 2003	Maffulli 2003 (a)	Maffulli 2003 (b)	Costa 2006	Kangas 2007	Suchak 2008	Groetelaers 2014
Khan 2004		**+**	**+**	**+**		**+**	**+**	**+**				
Khan 2005		**+**	**+**	**+**		**+**	**+**					
Suchak 2006		**+**	**+**		**+**	**+**	**+**	**+**				
Mark-Christensen 2016	**+**	**+**	**+**		**+**	**+**			**+**		**+**	
Huang 2015		**+**	**+**	**+**	**+**	**+**	**+**	**+**	**+**	**+**		
McCormack 2015		**+**	**+**	**+**	**+**	**+**	**+**	**+**	**+**	**+**	**+**	**+**

**Table 3:** Search database used by each study.

Author/Year	PubMed	Medline	Embase	Cochrane Library	CINAHL	Others
Khan 2004		+	+	+	+	+
Khan 2005		+	+	+	+	+
Suchak 2006		+	+	+	+	+
Mark-Christensen 2016	+		+	+	+	+
Huang 2015		+	+	+		+
McCormack 2015		+	+	+	+	+

**Table 4:** Methodological information for each included study.

Author/Year	Design of Included Studies	Level of Evidence	Software	GRADE Use	Subgroup Analysis
Khan 2004	RCT	Level I	Revman	No	No
Khan 2005	RCT	Level I	Revman	No	No
Suchak 2006	RCT	Level II	Revman	No	No
Mark-Christensen 2016	RCT	Level II	Revman	No	No
Huang 2015	RCT	Level II	Revman	No	Yes
McCormack 2015	RCT	Level I	Revman	No	No

RCT: Randomized clinical trial.

**Table 5:** AMSTAR criteria for each included study.

Items	Khan 2004	Khan 2005	Suchak 2006	Mark-Christensen 2016	Huang 2015	McCormack 2015
1. Was an a priori design provided?	1	0	0	0	0	0
2. Was there duplicate study selection and data extraction?	1	1	1	1	1	1
3. Was a comprehensive literature search performed?	1	1	1	1	1	1
4. Was the status of publication (i.e., grey literature) used as an inclusion criterion?	1	1	0	1	1	1
5. Was a list of studies (included and excluded) provided?	1	0	0	0	0	0
6. Were the characteristics of the included studies provided?	1	1	0	0	1	1
7. Was the scientific quality of the included studies assessed and documented?	1	1	1	1	1	1
8. Was the scientific quality of the included studies used appropriately in formulating conclusions?	1	0	1	1	0	0
9. Were the methods used to combine the findings of studies appropriate?	1	1	1	1	1	1
10. Was the likelihood of publication bias assessed?	0	0	0	0	0	0
11. Was the conflict of interest stated?	1	1	1	1	1	1
Total scores	10	7	6	7	7	7
